# Configurational and chain-mediational path-ways linking natural environment perception to restorative environmental perception

**DOI:** 10.1371/journal.pone.0343515

**Published:** 2026-03-06

**Authors:** Genmao Wang, Shuangquan Zhang

**Affiliations:** 1 Hunan Modern Logistics College, Changsha, China; 2 College of National Park and Tourism, Central South University of Forestry and Technology, Changsha, China; Dong-A University College of Business Administration, KOREA, REPUBLIC OF

## Abstract

While the link between natural environment perception (NEP) and restorative environmental perception (REP) is well-established, the specific psychological mechanisms—how environmental preference (EP) and place attachment (PA) configure this relationship—remain underexplored. This study aims to bridge this gap by examining the symmetric and asymmetric pathways translating nature perception into restorative outcomes. Analyzing survey data from 432 visitors to Zhangjiajie National Forest Park, China, we employed Partial Least Squares Structural Equation Modeling (PLS-SEM) to test serial mediation and Fuzzy-set Qualitative Comparative Analysis (fsQCA) to identify equifinal configurations. PLS-SEM results indicate that NEP enhances REP directly and indirectly through EP. Furthermore, a significant serial mediation path (NEP → EP → PA → REP) was established. Complementing these findings, fsQCA revealed EP as a “core condition” across all configurations for high REP, whereas PA serves as a “peripheral condition.” Theoretically, these results identify environmental preference as the critical gatekeeper for restoration. Practically, they suggest managers should prioritize aesthetics that trigger immediate preference to enhance restorative experiences.

## 1. Introduction

As the global population continues to grow and human activities increasingly encroach upon natural habitats, the effective management of natural resources has emerged as a critical priority. Protected areas, including national parks, forest parks, and other nature reserves, provide essential environmental services that significantly enhance human well-being. In the context of rising health challenges and escalating healthcare costs, public health authorities are increasingly turning to preventive strategies, recognizing the potential of natural environments as valuable resources for promoting health and preventing disease [1–3]. The therapeutic benefits of exposure to forest environments have garnered increasing attention in recent years [4]. The natural environment is now widely acknowledged to play a crucial role in both physical and mental health, with direct interaction with nature being essential for improving the overall quality of life [5]. The University of Washington’s “Green Cities: Health” initiative underscores the positive impact of nature on human health, synthesizing evidence from nearly 4,000 peer-reviewed studies that highlight the connection between nature and health. Similarly, Japan Medical University has established the Forest Medicine Research Society, which regularly publishes research on the link between forests and human health, aiming to raise public awareness of the substantial health benefits associated with forest environments.

Akgüller et al. (2025) [6] further confirmed that natural disasters, as extreme natural events, can significantly reshape the dynamics of economic sectors—their study on the Kahramanmaraş Earthquake showed that different economic sectors exhibit divergent resilience: some face increased volatility while others gain stability. This implies that natural environments not only provide direct health value but also interact with human economic systems through various pathways, underscoring the need to explore the multi-dimensional roles of natural spaces like forest parks. Batrancea et al. (2025) [7] revealed that “robustness to external shocks” can be systematically achieved through multi-dimensional optimization—by integrating uncertainty quantification and adaptive preferences, the framework mitigates crisis impacts while maintaining performance. This insight is highly relevant to forest park research: just as financial systems need robustness against market shocks, natural spaces like forest parks also require considering multi-dimensional demands (health/leisure/economic) to enhance resilience amid environmental or social changes. Batrancea et al. (2023) [8] confirms that wellbeing-related infrastructure—including natural environmental facilities like air quality and green spaces—is inherently interdependent with economic growth, as it enhances citizens’ wellbeing while driving regional economic vitality and mitigating disparities.

In an era where health needs are increasingly prominent, restoring physical and mental well-being has emerged as a primary motivation for travel [9]. Forest parks, designated as areas for travel, sightseeing, relaxation, and engagement in scientific and cultural activities, provide a unique setting within the natural forest environment. They offer aesthetic appeal, educational opportunities, and recreational benefits, all achieved through scientific conservation and thoughtful development. Forest parks are not only significant for their ecological and economic value but also for the rich psychological and physiological benefits they provide, extending beyond their recreational and educational functions. As crucial ecological resources and public goods, the value of forest parks is largely realized through ecotourism. To attract visitors, the development and design of forest parks must address current visitor needs while also adapting to evolving demands. This dual approach will maximize the parks’ functional value, enhance visitor experiences, and improve their competitiveness, thereby ensuring sustainable development.As interest in the restorative potential of forest parks continues to grow, scholarly attention has increasingly converged on this area of research. In 1983, Kaplan and colleagues first introduced the concept of a “Restorative Environment,” defining it as a setting that aids individuals in recovering from mental fatigue and stress-induced negative emotions. Kaplan further elaborated on this concept, describing restorative environments as spaces that facilitate the recovery from mental exhaustion and stress-related adverse emotional states. Environmental restoration refers to the degree to which an environment supports this recovery process, with higher levels of support being more conducive to initiating a restorative response [10]. Restorative environmental Perception (REP) emphasizes the subjective evaluation of an environment’s capacity to enable effective human recovery. The restorative effects of forest environments have been extensively documented, with visitors’ perceptions of these environments directly influencing their tourism satisfaction and, subsequently, their loyalty as visitors. In 1983, Ulrich introduced the Stress Reduction Theory, highlighting the benefits of interacting with natural environments. According to Ulrich, exposure to nature can rapidly enhance mood, with positive emotions fostering motivation, physiological balance, behavioral impulses, and adaptive responses, ultimately reducing both psychological and physiological stress [11]. Ulrich’s research suggests that the process of stress recovery involves not only healing from excessive psychological and physiological stress but also from under-stimulation or low levels of stress. It also includes replenishing the energy expended during the psychological or physiological responses to stress. Additionally, American psychologist Erich Fromm introduced the term biophilia in 1980, referring to the innate human tendency to be drawn to living things [12].

The notion of environmental preference, a cornerstone of environmental psychology, has garnered substantial research attention over the past five decades, resulting in a robust and well-structured theoretical foundation. Synthesizing both domestic and international definitions, environmental preference can be conceptualized as encompassing two primary dimensions: environmental cognition and environmental evaluation. It reflects the attitudes individuals hold toward specific environments, which subsequently shape their behavioral choices. The development of this preference begins with the initial observation of the environment, progresses through cognitive assessment and evaluation, and culminates in the formation of a preference [13]. Here, “preference” denotes the emotional response individuals exhibit toward various environmental stimuli, representing the ultimate evaluation of their affinity for a particular environment based on the dynamic interplay between the individual and their surroundings [14]. These preferences are forged through direct psychological responses, such as emotions, past experiences, and evolutionary principles, which can either attract or repel individuals from certain environments [15,16]. Environmental preference is inherently dynamic, influenced by a range of factors including past experiences, environmental familiarity, life stage, gender, education, and cultural background, all of which contribute to its variability over time [17].

Staats & Hartig (2004) [18] delve into the connection between environmental preferences and psychological needs, positing that restorative needs act as a catalyst for preference evaluations. They propose that individuals’ anticipatory judgments of an environment’s restorative capacities significantly shape their environmental preferences. For instance, natural environments are frequently favored due to their perceived ability to restore mental well-being. Variations in environmental preferences are particularly pronounced among individuals experiencing specific restorative needs, which can influence their preferences for either urban or natural settings. Furthermore, the degree to which an environment delivers on its restorative promise can refine these preferences. However, some studies, such as that by Roberts et al.(2021) [19], confirm that environments which are preferred can, in turn, become restorative. For example, museums can function as restorative spaces for regular visitors by facilitating self-regulation and stress reduction.Numerous studies have demonstrated that environmental preferences have a positive impact on perceptions of restorativeness, with higher preferences correlating with stronger restorative experiences [20]. Berg et al. (2003) [21] identified a positive relationship between individuals’ preference for a particular environment and its capacity to alleviate stress or mental fatigue, suggesting that those under stress or experiencing fatigue are more likely to benefit from the environment’s restorative qualities. Conversely, restorative perception has also been shown to influence environmental preference. Pals et al. (2009) [22], using the restorative environmental perception scale, found that dimensions such as charm, distance, and congruence are positively associated with environmental preference and can predict both preference and overall pleasantness. Liu et al. (2018) [23] constructed a path model examining the relationships among environmental preference, restorative perception, and health benefit assessment, using Fuzhou National Forest Park as their research site. Their findings indicated that the landscape environmental preference of visitors significantly enhances their restorative assessment.

The concept of place attachment, which originated in behavioral geography, has been widely studied globally and has garnered considerable attention in China since its introduction by scholars such as Huang Xiang in 2006. It is typically defined as the emotional bond between individuals and specific places, grounded in emotions, perceptions, and the passage of time [24]. Scannell & Gifford (2010) [25] reviewed numerous definitions and proposed a three-dimensional “person-cognitive process-place” framework to synthesize the concept of place attachment. In this framework, the person dimension reflects the perceived significance of the place to the individual; the cognitive process dimension encompasses the emotional, cognitive, and behavioral aspects of attachment; and the place dimension highlights the characteristics of the place itself, including the specificity of spatial elements and the importance of social or physical components. Place attachment enhances the psychological restorative effects of the environment, meaning that individuals’ emotional connections to aesthetically pleasing environments can aid in the recovery of physiological, psychological, and social capabilities that may have been depleted during adaptation to external stressors. Two primary theories explain the psychological restorative function of place attachment. The first is the Attention Restoration Theory, which emphasizes the environment’s role in restoring cognitive functioning. The second is the Psychological Evolution Theory, also known as the Stress Reduction Theory, which highlights the environment’s capacity to alleviate stress [26]. Place dependence influences all dimensions of restorative environmental perception, while place identity partially mediates these relationships. However, place identity affects dimensions of restorative environmental perceptions other than physical remoteness [27].

Despite the acknowledged benefits of forest therapy, a critical “black box” remains in understanding the cognitive-emotional processing that occurs between natural environments and human psychological restoration. Most studies assume a direct effect, yet environmental psychology suggests that human responses to landscape are mediated by evaluation (Preference) and emotional bonding (Attachment). Does a visitor need to like the forest for it to be restorative? Must they feel attached to it? The “person-cognitive process-place” framework suggests these are distinct steps. Furthermore, traditional linear regression models (like SEM) assume these relationships are uniform for all tourists. They fail to capture equifinality—the idea that different visitors might achieve restoration through different “recipes” of perception and emotion.

To address these gaps, this study integrates PLS-SEM and fsQCA to: (1) determine the mediating roles of Environmental Preference and Place Attachment; (2) test the serial mediation pathway; and (3) identify the configurational paths that lead to high restorative perception. This dual approach allows us to validate general mechanisms while respecting the complexity of individual tourist experiences. Notably, Akgüller et al. (2025) [28] proposed the fractional transfer entropy (FTE) framework, which captures complex interactive relationships by adjusting memory parameters and constructing directed weighted networks—an approach that resonates with the configurational thinking of fsQCA. Both methodologies emphasize moving beyond linear relationships to reveal multi-dimensional, context-dependent interaction patterns, providing a cross-disciplinary reference for analyzing the complex links between natural environments, human perceptions, and behavioral outcomes.

## 2. Literature review and hypotheses develop-ment

### 2.1. Natural environment perception and environmental preference

Environmental perception is an individual’s mental image of surroundings (and its formation/modification) and specifically the impression of environmental quality [29]. Existing studies rarely clarify this boundary, limiting empirical measurement precision. Environmental perception is essentially an individual-environment stimulus-response interaction leading to preference evaluations [30]. This model oversimplifies perception by ignoring moderating factors (experience, culture, individual differences), weakening its explanatory power. Kaplan (1977) [31] argued environmental preferences stem from human evolution, supported by tourists’ natural destination preference [32]. Yet, it overlooks contextual specificity, failing to explain urban preferences or socio-cultural influences—key gaps. Kaplan (1977) further linked aesthetic landscape preference to intrinsic “beauty” needs [31]. Undefined key concepts cause inconsistent empirical findings, hindering cross-study comparisons and applicability. Environmental preferences arise from human-environment interplay. Kaplan & Kaplan (1982) [33] identified four influencing factors, but their model neglects subjective cognition and lacks non-Western validation. Individuals prefer natural over man-made environments [34], with underlying mechanisms understudied—a gap addressed by the following hypotheses:

H1: Natural environment perception has a significant positive effect on environmental preference.

### 2.2. Natural environment perception and place attachment

In environmental perception research, behaviorists focus on consciousness formation. Downs’ “Conceptual Illustration of Geospatial Perception” states geographic landscapes shape environmental value systems [35], yet it frames perception as one-way, neglecting active subjective interpretation. Place attachment is a positive emotional bond to a place, characterized by proximity tendency [36]. It involves non-habitual environment ties [37], the desire to stay, safety feelings [36], and functional/emotional connections [38]. Evolving to a holistic emotion-cognition-behavior framework [39], its definitions lack clarity on habitual vs. non-habitual attachment boundaries. Landscapes elicit distinct perceptions, leading to place attachment for various reasons. While individuals may perceive attachment ambiguously, they favor connected places—yet literature fails to clarify social interactions’ moderating role. Consensus holds landscapes promote place attachment: a Norwegian study identified natural elements and family social life as key drivers; Kaltenborn & Bjerk (2002) [40] found place attachment enhances natural/historical landscape attractiveness. However, these studies have overlooked the role of natural environmental perception in place attachment across varying contextual conditions—a gap addressed by the following hypotheses:

H2: Natural environment perception has a significant positive impact on place attachment.

### 2.3. Environmental preference and place attachment

Environmental preference is an individual’s preference for a specific environment. Place attachment is based on environmental preference [41]: more frequent recreational evaluation of an environment increases reliance [42], and greater liking strengthens attachment [43]. Williams et al. (1992) [44] divided place attachment into functional place dependence (meeting user needs) and affective place identity (rooted in personal values and fostering belonging). Both reflect emotional connections to the environment. The biophilia hypothesis posits humans innately prefer open, low-risk environments, forming adaptive attachments [45]. Empirical studies support this: Kil et al. (2010) [46] found forest attachment correlates with forest preferences; Ryan (1997) [47] observed a moderate correlation between environmental preference and place attachment in urban parks/natural spaces. Place dependence also influences place identity, which may mediate its impact on environmental attitudes. However, existing studies have identified a moderate correlation but not its mechanism—a gap addressed by the following hypotheses:

H3: Environmental preference has a significant positive impact on place attachment.

### 2.4. Natural environment perception and restorative environmental perception

Natural environments have restorative qualities, alleviating stress-induced negative emotions and enhancing positive emotions [48–51]. Park et al. (2011) [52] confirmed forest environments regulate mood, promote relaxation, and reduce stress via questionnaires. A psychometric test on adult men showed forest exposure significantly boosted positive feelings and reduced negative emotions compared to urban stimuli [53,54], while natural scenes uniquely enhance subjective vitality [55]. Roszak et al. (1995) [56] found 90% of participants felt more energized outdoors; qualitative studies have examined vegetation-related resilience [57,58]. Mayer et al. (2009) [59] linked psychological recovery to nature connection, showing nature reserve walks yielded greater recovery via comparing students in natural and other settings. People in areas with favorable natural environments have lower mental stress and better coping abilities [60,61], and natural environments can mitigate job stress and reduce turnover intentions [62]. However, existing studies have obvious gaps: most focus on natural environments’ restorative effects but rarely clarify the direct link between natural environment perception and restorative environmental perception; few explore how natural environment perception affects different dimensions of restorative perception (e.g., compatibility, extent), and there is a lack of unified measurement standards, limiting cross-study comparisons and hypothesis validation—gaps addressed by the following hypotheses:

H4: Natural environment perception has a significant positive effect on compatibility.

H5: Natural environment perception has a significant positive effect on extent.

H6: Natural environment perception has a significant positive effect on mentally away.

H7: Natural environment perception has a significant positive effect on physically away.

H8: Natural environment perception has a significant positive effect on fascination.

### 2.5. Environmental preference and restorative environmental perception

Environmental preference refers to an individual’s selection of a favored environment. Studies have shown that people have unique environmental preferences (a human trait), and tend to have stronger emotional responses in preferred environments [63]. Environmental preferences derive from perceptual mechanisms that guide rapid automatic decisions to approach or avoid an environment, shaped by functionally significant environmental characteristics.There is a positive correlation between environmental preference and restoration: Hartig et al. (1997) found a 0.49 correlation between perceived environmental restoration and changes in environmental preference, indicating a significant association [64]. However, this is insufficient to confirm the mediating role of restoration effects in people’s preference for natural over built environments; without considering environmental type, the relationship between environmental type and preference may be spurious (mirroring that between restorative potential and preference). Testing mediation requires verifying the link between restorative potential (or actual restoration) and preference after statistically controlling for environmental type’s influence on preference. Existing studies also have gaps: few clarify how environmental preference, as a derivative of natural environment perception, affects different dimensions of restorative perception, and the spurious correlation issue mentioned above lacks in-depth verification—gaps addressed by the following hypotheses:

H9: Environmental preference has significant positive effect on compatibility

H10: Environmental preference has a significant positive effect on extent

H11: Environmental preference has a significant positive effect on mentally away

H12: Environmental preference has a significant positive effect on physically away

H13: Environmental preference has a significant positive effect on fascination

### 2.6. Place attachment and restorative environmental perception

Place attachment, an outcome of spatial experience, is associated with tourists’ psychological recovery and environmental restorative perception. You et al. (2018) [65] explored the relationship between place attachment emotions and forest park visitors’ restorative experiences. Existing studies confirm place attachment positively affects restorative experiences, as individuals relax and express themselves more freely in attached places, achieving greater recovery. Ratcliffe & Korpela (2016) [66] found place memory, place attachment, and place identity positively impact such perception. Place attachment enables place memory to promote psychological recovery, and place identity directly affects psychological recovery [67], with place attachment also positively influencing environmental restorative perception [68]. Additionally, Wilkie & Clouston (2015) [69] found urban residents engaging in recreation in preference-aligned, high-local-identity environments experience more restorative characteristics; Bornioli et al. (2018) [70] noted stronger city identity correlates with higher ratings of restorative perception’s distance and fascination dimensions. However, few existing studies have elucidated how place attachment (as a key emotional bond) influences multiple dimensions of restorative environmental perception, and the verification of differences in this relationship within natural environmental contexts remains insufficient—gaps addressed by the following mediating hypotheses:

H14: Place attachment has a significant positive effect on compatibility.

H15: Place attachment has a significant positive effect on extent.

H16: Place attachment has a significant positive effect on mentally away.

H17: Place attachment has a significant positive effect on physically away.

H18: Place attachment has a significant positive effect on fascination.

### 2.7. The mediating role of environmental preference

Natural tourism destinations with beautiful, eco-friendly environments can enhance physical and mental illness treatment, function reconstruction, and overall health recovery. Researchers have extensively studied the restorative potential of natural environments (e.g., parks, beaches, green spaces) [71]; some have also explored urban man-made environments, finding museums [72], zoos [22], and shopping malls [73] possess restorative properties, though natural environments generally have greater restorative potential [74]. The sensory dimensions of environmental perception can elicit visitor preferences and trigger restorative responses, which can improve mental health promotion effectiveness in forest park design [75]. However, existing studies have obvious gaps: few explicitly examine the mediating role of environmental preference between natural environment perception and the dimensions of environmental restorative perception, and the mechanism by which natural environment perception influences restorative perception through environmental preference lacks in-depth verification—gaps addressed by the following mediating hypotheses:

H19: Environmental preference mediates between natural environment perception and compatibility.

H20: Environmental preference mediates between natural environment perception and extent.

H21: Environmental preference mediates between natural environment perception and mentally away.

H22: Environmental preference mediates between natural environment perception and physically away.

H23: Environmental preference mediates between natural environment perception and fascination.

### 2.8. The mediating role of place attachment

Natural environmental features significantly contribute to tourists’ place attachment formation; urban residents’ perception of natural changes in their living environment positively influences their place attachment. Various studies confirm the natural environment is a key factor in place attachment development: Chi & Su (2012) [76] found the restorative effect of natural environment images (emotional priming and focused attention restoration) depends on individuals’attachment level to the environment—only attached environments have attention-restoring and emotion-initiating effects, while low-attachment natural environments may elicit negative emotions. Research on place attachment’s mediating role in environmental preference and restorative perception remains underdeveloped, requiring more empirical verification. Moore & Graef (1994) [42] showed urban residents’ community functional dependence positively impacts emotional attachment, with place dependence influencing environmental attitudes and behaviors via place identity’s mediation. Bornioli et al. (2018) [70] also found people’s memories of favorite places indirectly affect environmental restorative perception through place attachment’s mediation. However, existing studies have gaps: few explicitly examine the mediating role of place attachment between natural environment perception and the five dimensions of environmental restorative perception, and the internal mechanism of this mediation process lacks in-depth exploration—gaps addressed by the following hypotheses:

H24: Place attachment mediates between natural environment perception and compatibility.

H25: Place attachment mediates between natural environment perception and extent.

H26: Place attachment mediates between natural environment perception and mentally away.

H27: Place attachment mediates between natural environment perception and physically away.

H28: Place attachment mediates between natural environment perception and fascination.

### 2.9. Chain mediation of environmental preference and place attachment

Preferred environments are more likely to be perceived as restorative. Studies have confirmed a link between place attachment and preference: restorative experiences enhance environmental preference, which in turn promotes place attachment [77]. Liu et al. (2018) [23] constructed an urban park restorative evaluation psychological model, empirically verifying the mechanism of environmental preference and place attachment on restorative perception via Fuzhou urban parks, confirming place attachment’s mediating role and proposing the sequence: “environmental preference → place dependence → place identity → restorative perception.” Natural environments easily trigger environmental preference; stronger preference correlates with higher restorative perception, and preference positively affects place attachment, which further influences restorative perception. Thus, natural environment perception may indirectly affect restorative perception through the chain mediation of environmental preference and place attachment. However, existing studies have gaps: few explicitly examine the chain mediating role of environmental preference and place attachment between natural environment perception and the five dimensions of environmental restorative perception, and the internal sequential mechanism of this chain mediation remains understudied—gaps addressed by the following hypotheses:

H29: Environmental preference and place attachment mediate between natural environment perception and compatibility.

H30: Environmental preference and place attachment mediate between natural environment perception and extent.

H31: Environmental preference and place attachment mediate between natural environment perception and mentally away.

H32: Environmental preference and place attachment mediate between natural environment perception and physically away.

H33: Environmental preference and place attachment mediate between natural environment perception and fascination.

**Fig 1:**
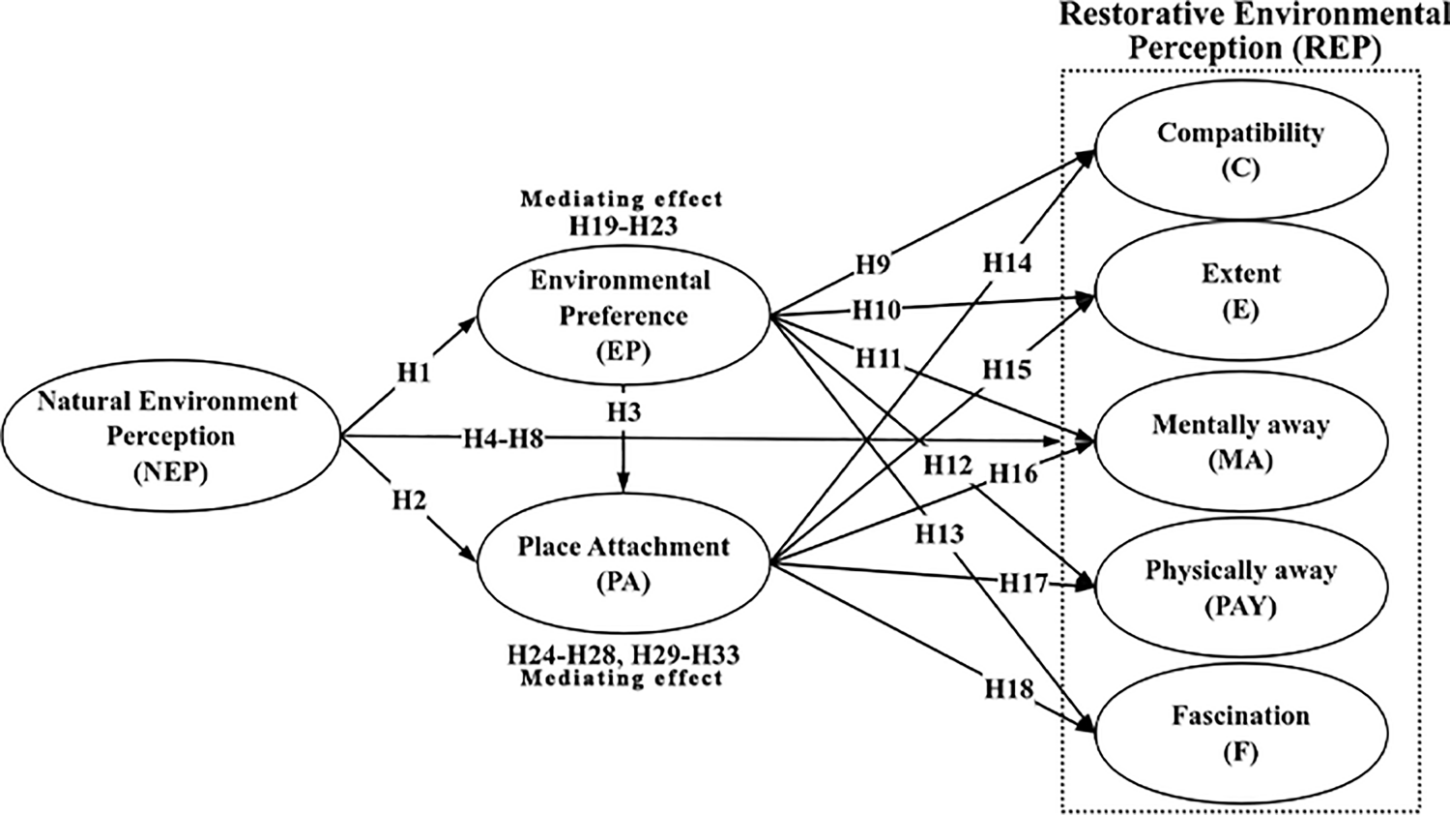
Conceptual model.

Based on the research hypotheses, the conceptual model of this study was constructed as shown in Fig 1.

## 3. Materials and methods

### 3.1. Study site

**Fig 2:**
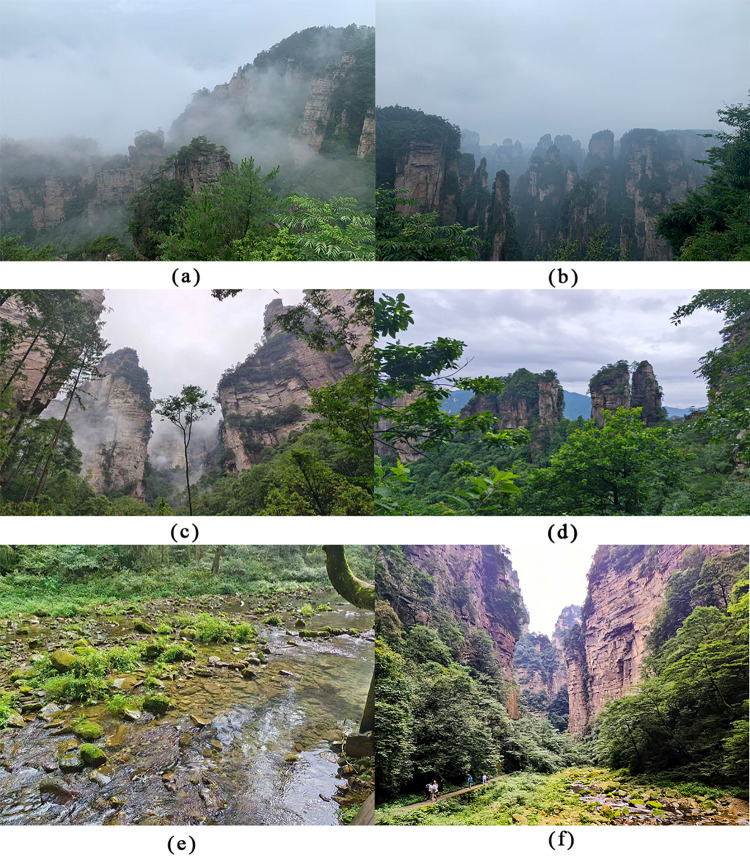
Zhangjiajie National Forest Park (Author’s own photos).

In this study, Zhangjiajie National Forest Park (Fig 2), which aligns closely with the study’s objectives, was selected as the research site. An empirical study was conducted by collecting relevant data from tourists through field surveys. The reasons for choosing this site are as follows: (1) Zhangjiajie National Forest Park, located in the Wulingyuan mountain range of Zhangjiajie City, covers an area of 130 square kilometers and is the first national forest park in China. In December 1992, the park was designated as a UNESCO World Nat-ural Heritage site due to its unique quartz sandstone Great Peak Forest. The park boasts abundant forest resources, including 517 species of woody plants. It is a rare tourist destination, offering opportunities for relaxation and immersion in nature, thanks to its breathtaking scenery and diverse flora and fauna in the high mountains and gorges. (2) The park attracts numerous domestic and international tourists due to its distinctive natural beauty. In 2023, the cumulative number of visitors to Zhangjiajie National Forest Park reached 7,012,000, with a peak of 4,872,000 ticket purchases at one time. This represents a year-on-year increase of 233.4% and a 13.4% increase compared to 2019. Given the high visitor volume, this site was chosen for the questionnaire research to gather a larger sample of tourist data, thereby enhancing the credibility of the study’s findings.

### 3.2. Measurement items

The natural environment perception scale comprised seven items, the environmental preference scale contained four items, and the place attachment scale consisted of seven items adapted from Williams and Vaske, covering the dimensions of place dependence and place identity. The restorative environmental perception scale included 18 items. All scales employed a Likert 5-point format, with responses ranging from “strongly disagree” (1) to “strongly agree” (5). A higher score indicates a stronger perception of natural environment, environ-mental preference, place attachment, and restorative environment perception. The sources of scale indicators are detailed in Table 1.

**Table 1:** Measurement items and sources.

Latent variables	Variable quantity	Observable items	References
Natural environment perception (NEP)	NEP1	This area has a greater amount of biological landscapes	Kaltenborn et al.[78] Wang et al.[79] Zheng et al.[80] Liu et al.[63] Tenngart Ivarsson et al.[81] Zung [82] Hartig et al.[64] Lehto et al.[83] Hartig et al. [84]
	NEP2	The forest cover in this location is diverse and layered	
	NEP3	Celestial and meteorological landscapes that are both magnificent and varied	
	NEP4	The water body is eroding and the natural environment of the area it flows through is highly diverse	
	NEP5	Forested vegetation encompasses a wide range of diversity and variety	
	NEP6	The terrain is characterized by its diversity and variety	
	NEP7	The road traverses an area with a diverse natural environment	
Environmental preference (EP)	EP1	The forested landscape here is continuous	Kaplan et al.[85] Huang et al.[32]
	EP2	The forested landscape here is made up of layers	
	EP3	The forested landscape here is full of changes	
	EP4	The forested landscape here is rich and diverse	
Place attachment (PA)	PA1	This place is ideal for what I want to do	Williams et al.[86] Fan et al.[87] Liu et al.[63]
	PA2	This place is unlike any other	
	PA3	This trip is significant to me	
	PA4	It’s more crucial to do things here than to do the same thing elsewhere	
	PA5	This place has provided me with an experience that cannot be matched anywhere else	
	PA6	This place holds a special meaning for me	
	PA7	I am a huge admirer of this lifestyle	
Compatibility (C)	C1	This place makes me feel at ease	Hartig et al.[64] Tenngart Ivarsson et al.[81] Zung [82] Lehto et al.[83]
	C2	This place is filled with friendliness	
	C3	I am in harmony with nature	
	C4	The overall environment is in harmony with everything here	
	C5	This is a place where I can do the things I desire and enjoy	
Extent (E)	E1	This place offers me a variety of activities that I can participate in	Tenngart Ivarsson et al.[81] Lehto et al.[83] Laumann et al.[88]
	E2	I have the ability to do a lot of things here that I normally cannot, but I am extremely intrigued by them	
	E3	I can participate in different activities, programs, and experiences in different areas of the site	
	E4	Exploring in many directions is possible due to the ample space and area	
Mentally away (MA)	MA1	I can unwind from my responsibilities and pressures for a while here	Hartig et al.[64] Laumann et al.[88]
	MA2	I can take a break from the constant routine of daily life here	
Physically away (PAY)	PAY1	It feels like i am in a different setting than what I am used to	Lehto et al.[83] Laumann et al.[88]
	PAY2	The activities I engage in here are not the same as those I engage in at home	
	PAY3	The environment I live in every day is very different from this one	
Fascination (F)	F1	It’s captivating and unforgettable	Laumann et al.[88] Tenngart Ivarsson et al.[81] Hartig et al.[64]
	F2	There are numerous fascinating things to discover and explore here	
	F3	It has a lot of charm	
	F4	This place is going to be deeply missed by me	

### 3.3. Data collection

In this study, data was collected by randomly distributing paper questionnaires, and tourists participated in the survey and answered the questionnaires independently. The study used only anonymous, non-sensitive questionnaires and posed no more than minimal risk; data collection was carried out in the everyday flow of visitors. Because requesting written signatures would have created a record of identity and increased participants’ psychological burden, consent was obtained orally. Before handing out the questionnaire, the interviewer explained the study purpose, procedures, anonymity safeguards, and the right to withdraw at any time to each potential participant. After explicit verbal consent was given, the interviewer ticked a box on the front page of the questionnaire to document consent, and a member of the research team co-signed as a witness. The Academic Committee approved the use of verbal informed consent on the grounds that the study involved low risk and that obtaining written consent was imprac-tical. The questionnaires were distributed from June 22, 2024 to June 25, 2024. 490 questionnaires were distributed, 460 were recovered, and 432 were valid. The recovery rate was 94%. Table 2 exhibits the basic information of the respondents.

**Table 2:** Demographic profile of respondents.

Indicator	Item	Frequency	%
Gender	Female	196	45.37
	Male	236	54.63
Age	18 to 24 years old	171	39.58
	25 to 34 years old	111	25.69
	35 to 44 years old	66	15.28
	45 to 54 years old	49	11.34
	55 to 64 years old	28	6.48
	65 to 74 years old	6	1.39
	75 and above	1	0.23
Education	Junior high school and below	63	14.58
	High school and vocational school	106	24.54
	Junior College and Undergraduate	210	48.61
	Graduate student and above	53	12.27
Annual personal income	24,000 RMB below	181	41.90
	24,000–60,000 RMB	104	24.07
	60,000–120,000 RMB	99	22.92
	Above 120,000 RMB	48	11.11
Job	National civil servants	15	3.47
	Enterprise and public utility managers	31	7.18
	Unit staff/workers	60	13.89
	Private Owners	39	9.03
	Military personnel	8	1.85
	Unemployed/Laid-off	8	1.85
	Professionals and technicians (e.g., doctors, teachers)	28	6.48
	Farmers	11	2.55
	Students	180	41.67
	Retirees	12	2.78
	Others	40	9.26
Visiting times	1 time	291	67.36
	2 times	61	14.12
	3 times	23	5.32
	4 times	10	2.31
	5 times and above	47	10.88

In terms of gender, among the 432 tourists in this survey, there is a small difference between the number of male and female tourists, which is in line with the requirements of this questionnaire; in terms of age distribution, the largest proportion in this survey is 18–24 years old, accounting for 39.58%. People aged 25–34 and 35–44 come in second, with 25.69% and 15.28% respectively. In terms of education, the proportion of college degree and bachelor’s degree is the highest, at 48.61%, indicating that the population in this survey is generally better educated; in terms of jobs, the proportion of students and unit staff/workers is higher, at 41.67% and 13.89% respectively; in terms of annual personal income, people with less than 24,000 yuan predominate, followed by 24,000–60,000 and 60,000–120,000, while the proportion of other incomes is relatively balanced, which indicates that the economic incomes of the surveyed population are lower than the average level, which is mainly due to the fact that the surveyed popula-tion is mainly composed of students and unit workers. Regarding the number of visiting times, tourists who came to Zhangjiajie National Forest Park for the first time were in the majority, accounting for 67.36% of all tourists, followed by those who came for the second time and the fifth time and above, accounting for 14.12% and 10.88% respectively

### 3.4. Methodology

This study utilized SPSS 21.0 and SPSSAU software to examine the com-mon method bias and total reliability of the questionnaire. Validated factor analysis (CFA) was used to test the structural, convergent, and discriminant validity of the questionnaire. SmartPLS 4.0 was used to perform PLS-SEM analysis and to test the mediation effect. FsQCA 3.0 was employed for configurational analysis. The research protocol was reviewed and approved by the Academic Committee of Central South University of Forestry and Technology. All participants provided informed verbal consent prior to inclusion in the study. Written consent was waived by the committee to ensure respondent anonymity and to facilitate data collection in a high-flow tourist environment where collecting signatures would impede the naturalistic setting. The investigator read a standardized script explaining the study purpose, anonymity, and right to withdraw, and checked a box on the questionnaire to document participant assent.

## 4. Results

### 4.1. Common method bias test

To avoid bias in data analysis and ensure compliance with relevant normative requirements before conducting quantitative analysis, the Harman one-way test was used to assess common method bias in the sample. This test involved performing an unrotated factor analysis on all the measured variables. If a single factor did not account for the majority of the variance, common method bias would be considered negligible [89]. The unrotated factor analysis of all variables in the scale revealed that the first principal component explained 28.761% of the variance, and the total explained variance was 60.551%. Additionally, the highest correlation coefficient among all variables was 0.669, which is below the threshold of 0.9. Therefore, it was determined that common method bias was not a significant concern in this study.

### 4.2. Reliability and validity tests

The total reliability of the questionnaire was tested using SPSS 21.0 to obtain its Cronbach’s Alpha value, if this value is higher than 0.8, it indicates high reliability, if this value is between 0.7–0.8, it indicates good reliability, if this value is between 0.6–0.7, it indicates acceptable reliability and if this value is less than 0.6, it indicates poor reliability. The analysis shows that Cronbach’s Alpha value = 0.940, which indicates that the questionnaire is highly reliable. The validity of the questionnaire scale should be tested in four aspects: content validity, criterion validity, construct validity and conjoint validity [90]. The criterion validity coefficient is commonly used to evaluate criterion validity, but it is not typically reported in tests because it is difficult to calculate and use [91]. The construction of the scale in this paper is mainly based on the existing research results at home and abroad, interviews with tourists and three rounds of expert opinion solicitation, and the indicators are screened through scientific methods, so the scale has high content validity. The degree and ability of the questionnaire scale’s content to measure theoretical abstract concepts is referred to as construct validity. The usual practice is to use the variance contribution of the first principal component of each latent variable to determine the variance. A general requirement of more than 40% is acceptable [91]. The test of convergent and discriminant validity of a questionnaire scale is known as conjoint validity. Convergent validity is tested by analyzing the standardized loading coefficients of each measurement item of the latent variable and extracting the average variance (AVE) [92]. The closer the standardized factor loadings are to 1, the better the effect is In this study, the standardized factor loadings of 0.6 were used as the criterion for the exclusion of indicators in the validation factor analysis stage. When the AVE value of each latent variable is greater than 0.5 and the standardized factor loading is greater than 0.6, the measurement question items can explain most of the variance variance of each latent variable, indicating that the scale has good convergent validity. Discriminant validity was determined by comparing the square root of the mean variance extracted from the latent variables with the absolute value of the correlation coefficients between the variables, and if the former was greater than the latter, it indicated that the variables had good discriminant validity [93]. The AVE and CR scores of the model are used to measure the convergent validity of the pairs of variables within a factor, and an AVE higher than 0.4 or a CR higher than 0.7 usually indicates high convergent validity.

Table 3 presents the descriptive statistics for the measurement items, including mean, standard deviation (SD), skewness, and kurtosis. The mean values for the constructs generally ranged from 3.60 to 4.56, indicating a positive reception of the natural environment and restorative qualities by the respondents. The standard deviations ranged between 0.63 and 1.09, reflecting a reasonable level of variability in the responses.The skewness values ranged from −1.51 to −0.25, and kurtosis values were largely within the acceptable range of −1 to +3. Although PLS-SEM is a non-parametric method and does not strictly require normal distribution, these results suggest that the data does not deviate severely from normality, ensuring the robustness of the parameter estimates.

**Table 3:** Descriptive statistics and normality test.

Construct	Item	Mean	SD	Skewness	Kurtosis	VIF (Outer)
**Natural Environment Perception**	NEP1	4.56	0.671	−1.513	2.275	2.14
	NEP2	4.55	0.633	−1.211	0.842	2.31
	NEP3	4.51	0.667	−1.296	1.835	2.08
	NEP4	4.48	0.690	−1.083	0.377	1.95
	NEP5	4.51	0.687	−1.460	2.656	2.47
	NEP6	4.49	0.694	−1.328	1.854	2.22
	NEP7	4.53	0.680	−1.423	2.105	2.36
**Environmental Preference**	EP1	4.38	0.748	−1.343	2.708	1.89
	EP2	4.35	0.733	−0.795	−0.030	1.76
	EP3	4.34	0.743	−0.807	−0.169	1.82
	EP4	4.41	0.707	−1.000	0.549	1.94
**Place Attachment**	PA1	3.82	0.998	−0.579	−0.058	2.11
	PA2	3.60	1.091	−0.250	−0.843	1.98
	PA3	3.72	1.040	−0.417	−0.456	2.05
	PA4	3.88	0.987	−0.529	−0.505	2.23
	PA5	4.06	0.897	−0.751	0.192	2.17
	PA6	4.33	0.852	−0.920	0.650	1.85
	PA7	4.10	0.869	−0.700	−0.041	1.92
**Compatibility**	C1	4.39	0.702	−1.119	1.823	2.45
	C2	4.29	0.788	−1.012	1.088	2.33
	C3	4.36	0.703	−0.800	0.009	2.28
	C4	4.35	0.733	−0.896	0.462	2.39
	C5	4.18	0.856	−0.871	0.412	2.15
**Extent**	E1	3.99	0.878	−0.513	−0.222	1.78
	E2	4.07	0.894	−0.769	0.223	1.84
	E3	4.15	0.858	−0.939	0.798	1.96
	E4	4.41	0.758	−1.315	1.876	1.89
**Mentally Away**	MA1	4.40	0.796	−1.454	2.427	2.65
	MA2	4.40	0.772	−1.252	1.465	2.71
**Physically Away**	PAY1	4.41	0.724	−1.249	1.960	2.54
	PAY2	4.42	0.683	−1.247	2.562	2.68
	PAY3	4.41	0.707	−1.197	2.014	2.59
**Fascination**	F1	4.41	0.681	−0.949	0.834	2.41
	F2	4.37	0.765	−0.959	0.082	2.26
	F3	4.42	0.715	−1.152	1.339	2.38
	F4	4.25	0.821	−1.075	1.283	2.12

Furthermore, lateral collinearity was assessed to ensure that common method bias or high correlations between predictors did not distort the results. As indicated in Table 3, the Variance Inflation Factor (VIF) values for all items ranged from 1.76 to 2.71. All values are well below the conservative threshold of 3.0 (and the lenient threshold of 5.0), confirming that multicollinearity is not a significant issue in this study. This validates the distinctiveness of the constructs and supports the structural model’s reliability.

According to Table 4, a total of eight factors corresponding to the combined reliability CR values are greater than 0.7, which can indicate that the questionnaire scales have high convergent validity, indicating that the extraction of the measures within the factors is more excellent.

**Table 4:** Confirmatory factor analysis.

Variables	Variable quantity	Standard error	p	Standardized factor loading	CR	AVE
Natural Environment Perception	NEP1	–	–	0.656	0.873	0.496
	NEP2	0.079	0.000	0.676		
	NEP3	0.084	0.000	0.714		
	NEP4	0.087	0.000	0.713		
	NEP5	0.087	0.000	0.724		
	NEP6	0.087	0.000	0.685		
	NEP7	0.086	0.000	0.758		
Environmental Preference	EP1	–	–	0.701	0.782	0.473
	EP2	0.074	0.000	0.736		
	EP3	0.074	0.000	0.642		
	EP4	0.071	0.000	0.668		
Place Attachment	PA1	–	–	0.734	0.848	0.459
	PA2	0.075	0.000	0.743		
	PA3	0.060	0.000	0.621		
	PA4	0.071	0.000	0.817		
	PA5	0.068	0.000	0.726		
	PA6	0.062	0.000	0.678		
	PA7	0.179	0.000	0.283		
Compatibility	C1	–	–	0.739	0.846	0.523
	C2	0.074	0.000	0.726		
	C3	0.066	0.000	0.725		
	C4	0.069	0.000	0.720		
	C5	0.081	0.000	0.704		
Extent	E1	–	–	0.715	0.834	0.559
	E2	0.074	0.000	0.797		
	E3	0.071	0.000	0.799		
	E4	0.062	0.000	0.671		
Mentally away	MA1	–	–	0.759	0.758	0.610
	MA2	0.067	0.000	0.803		
Physically away	P1	–	–	0.714	0.797	0.568
	P2	0.072	0.000	0.817		
	P3	0.073	0.000	0.726		
Fascination	F1	–	–	0.811	0.843	0.575
	F2	0.062	0.000	0.753		
	F3	0.057	0.000	0.779		
	F4	0.068	0.000	0.683		

Note: A ‘-’ indicates that the item is a reference item.

As can be seen from Table 5, the bolded values in the graph are AVE square root values, which are used to measure the discriminant validity of the scale, if the AVE square root value of one of the factors is greater than the correlation coefficient between it and the other factors, then it means that this factor has a more excellent discriminant validity. For example, the AVE square root value of place attachment in the table is 0.677, which is greater than the absolute maximum value of the correlation coefficient between the factors, 0.563, which means that it has good discriminant validity.

**Table 5:** Pearson correlation and the square root of AVE.

Variables	Natural Environment Perception	Environmental Preference	Place Attachment	Compatibility	Extent	Mentally away	Physically away	Fascination
**Natural Environment Perception**	**0.704**							
**Environmental Preference**	0.690	**0.688**						
**Place Attachment**	0.379	0.440	**0.677**					
**Compatibility**	0.616	0.674	0.520	**0.723**				
**Extent**	0.527	0.595	0.563	0.703	**0.747**			
**Mentally away**	0.568	0.568	0.467	0.634	0.626	**0.781**		
**Physically away**	0.527	0.522	0.384	0.612	0.538	0.577	**0.754**	
**Fascination**	0.631	0.619	0.512	0.684	0.625	0.597	0.651	**0.758**

Note: Bolded numbers are AVE square root values.

### 4.3. Structural equation modeling test

Based on the established fit criteria, an SRMR < 0.05 indicates an ideal model fit, whereas d_ULS < 0.95 and d_G < 0.95 signify good model fit [94]. The empirical results show that the model achieves an SRMR of 0.033, a d_ULS of 0.719, and a d_G of 0.422, all of which satisfy the recommended thresholds, thus confirming that the structural equation model demonstrates a satisfactory fit to the data.

**Fig 3:**
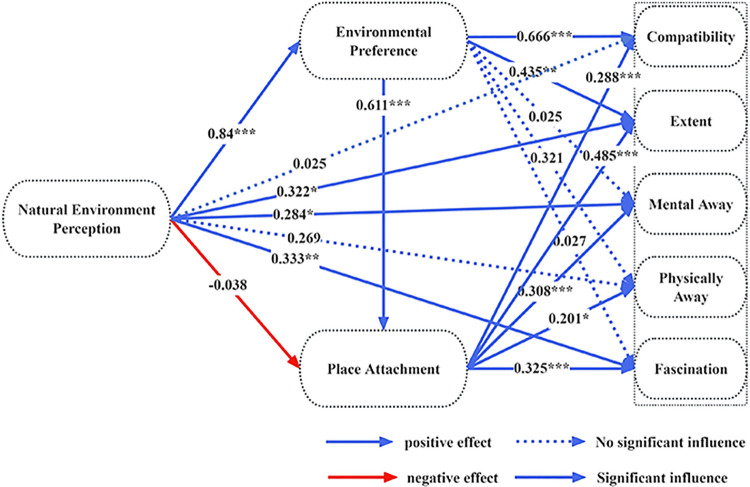
PLS-SEM output results.

Meanwhile, the relationships among Natural Environment Perception (NEP), Environmental Preference (EP), Place Attachment (PA), and Restorative Environmental Perception (REP) were further examined via structural-equation modeling; the results are reported in Table 6 and illustrated in Fig 3. Of the 18 hypothesized paths, 5 were non-significant – namely, Natural Environment Perception (NEP) → Place Attachment (PA), Natural Environment Perception (NEP)→Compatibility (C), Natural Environment Perception (NEP) →Extent (E), Natural Environment Perception (NEP) → Physically away (PAY), and Environmental Preference (EP) →Physically away (PAY) – leading to the rejection of hypotheses H2, H4, H5, H7, and H12. The remaining 13 hypotheses received empirical support.

**Table 6:** Results of model output.

Hypothesis	Path	Standard Error	T-statistic	P-value	Test Result
H1	NEP - > EP	0.032	26.478	0.000	Supported
H2	NEP - > PA	0.146	0.261	0.794	Not Supported
H3	EP - > PA	0.149	4.095	0.000	Supported
H4	NEP - > C	0.116	0.219	0.826	Not Supported
H5	NEP - > E	0.113	0.237	0.813	Not Supported
H6	NEP - > MA	0.130	2.191	0.029	Supported
H7	NEP - > PAY	0.149	1.806	0.071	Not Supported
H8	NEP - > F	0.121	2.761	0.006	Supported
H9	EP - > C	0.134	4.962	0.000	Supported
H10	EP - > E	0.139	3.139	0.002	Supported
H11	EP - > MA	0.146	2.209	0.027	Supported
H12	EP - > PAY	0.181	1.773	0.076	Supported
H13	EP - > F	0.141	2.118	0.034	Supported
H14	PA - > C	0.065	4.433	0.000	Supported
H15	PA - > E	0.070	6.968	0.000	Supported
H16	PA - > MA	0.065	4.750	0.000	Supported
H17	PA - > PAY	0.084	2.394	0.017	Supported
H18	PA - > F	0.064	5.095	0.000	Supported

### 4.4. Mediation effect test

As can be seen from Table 7, the mediating roles of Place Attachment (PA) between Natural Environment Perception (NEP) and Compatibility (C), Natural Environment Perception (NEP) and Extent (E), Natural Environment Perception (NEP) and Mentally away (MA), and Natural Environment Perception (NEP) and Fascination (F) are not significant, so Hypothesis H24, H25, H26 and H28 are invalid. Furthermore, the mediating role of Environmental Preference (EP) in the relationship between Natural Environment Perception (NEP) and Physically away (PAY) was not significant; thus, Hypothesis H22 is rejected. The remaining ten hypotheses concerning mediation effects are empirically supported.

**Table 7:** Mediation effect test results.

Hypothesis	Path	Standard Error	T-statistic	P-value	Test Result
H19	NEP - > EP - > C	0.123	4.547	0.000	Supported
H20	NEP - > EP - > E	0.122	2.999	0.003	Supported
H21	NEP - > EP - > MA	0.126	2.148	0.032	Supported
H22	NEP - > EP - > PAY	0.155	1.744	0.081	Not Supported
H23	NEP - > EP - > F	0.121	2.070	0.038	Supported
H24	NEP - > PA - > C	0.043	0.255	0.799	Not Supported
H25	NEP - > PA - > E	0.071	0.260	0.795	Not Supported
H26	NEP - > PA - > MA	0.046	0.257	0.797	Not Supported
H27	NEP - > PA - > PAY	0.033	0.234	0.815	Not Supported
H28	NEP - > PA - > F	0.050	0.247	0.805	Not Supported
H29	NEP - > EP - > PA - > C	0.047	3.116	0.002	Supported
H30	NEP - > EP - > PA - > E	0.068	3.665	0.000	Supported
H31	NEP - > EP - > PA - > MA	0.053	2.968	0.003	Supported
H32	NEP - > EP - > PA - > PAY	0.051	2.010	0.044	Supported
H33	NEP - > EP - > PA - > F	0.059	2.835	0.005	Supported

### 4.5. Multiple‐path analysis based on fsQCA

#### 4.5.1. Variable selection and data calibration.

The PLS-SEM results indicate that Natural Environment Perception, Environmental Preference, and Place Attachment exert significant effects on all five dimensions of Restorative Environmental Perception. Yet it remains unclear whether these three antecedents operate configurationally and which specific combinations of conditions are sufficient for high Restorative Environmental Perception. We therefore retain these factors as the causal conditions for the subsequent fsQCA, aiming to uncover their joint effects from a configurational perspective. Prior to the fsQCA, the survey data must be transformed into fuzzy sets with membership scores ranging from 0 to 1 to enhance interpretability. Because fsQCA requires unidirectional variables, multi-item constructs were first reduced to a single score by averaging their respective indicators. Following Ragin’s guidelines, three qualitative anchors were specified: the 5th percentile, the 95th percentile, and the 50th percentile [95]. Table 8 presents the fuzzy-set calibration anchors for all conditions.

**Table 8:** Calibration anchors for all constructs.

Constructs		Calibration anchors		
		95% (fully in)	50% (cross-over)	5% (fully out)
Conditions	Natural Environment Perception	5	4.7143	3.5714
	Environmental Preference	5	4.5	3.25
	Place Attachment	5	3.8333	2.5
Outcomes	Compatibility	5	4.4	3.2
	Extent	5	4.25	3
	Mentally away	5	4.5	3
	Physically away	5	4.6667	3.3333
	Fascination	5	4.5	3

#### 4.5.2. Necessity analysis of single conditions.

Prior to examining configurational solutions, we conducted a necessity analysis with fsQCA 3.0 to determine whether any individual antecedent condition is required for the outcome Restorative Environmental Perception. Following Fiss [96], a condition is deemed necessary when its consistency score exceeds 0.9. The results, reported in Table 9, show that all three single conditions—Natural Environment Perception, Environmental Preference, and Place Attachment—surpass this threshold. Consequently, each condition constitutes a necessary prerequisite for high Restorative Environmental Perception and must be retained in the subsequent sufficiency analysis.

**Table 9:** Necessity analysis of antecedent conditions for restorative environmental perception.

Outcome	Conditions	Consistency	Coverage
Compatibility	Natural Environment Perception	1	0.119386
	Environmental Preference	1	0.123471
	Place Attachment	1	0.138250
Extent	Natural Environment Perception	1	0.115358
	Environmental Preference	1	0.119306
	Place Attachment	1	0.133586
Mentally away	Natural Environment Perception	1	0.129636
	Environmental Preference	1	0.134072
	Place Attachment	1	0.150119
Physically away	Natural Environment Perception	1	0.112048
	Environmental Preference	1	0.115882
	Place Attachment	1	0.129752
Fascination	Natural Environment Perception	1	0.122195
	Environmental Preference	1	0.126376
	Place Attachment	1	0.141502

Note: Consistency and Coverage are key fit indices in fsQCA, reflecting the relationship between each condition and the corresponding outcome.

#### 4.5.3. Configurational analysis: Sufficiency and solution types.

Given the medium-N sample of 432 valid questionnaires, we adhered to Schneider and Wagemann’s [97] guideline that at least 75% of all empirically possible cases should be retained. Inspection of the truth-table distribution led to a frequency threshold of 7; the results for each outcome are reported in Table 10. fsQCA 3.0 generated three solution types—complex, parsimonious, and intermediate. Following current best practice, we report the intermediate solutions and use the parsimonious solutions to identify core conditions. All individual configurations and the overall solutions exceed the 0.75 consistency benchmark recommended for sufficiency analyses (Ragin, 2008).

**Table 10:** Configurational paths of antecedents leading to restorative environmental perception.

Antecedent Conditions	Configurations								
	1	2	1	2	1	2	1	1	2
Natural Environment Perception		●	⊗	●	●	●	●	●	
Environmental Preference	●		●			●	●		●
Place Attachment		●		●			●		
Consistency	0.838996	0.892418	0.861685	0.873300	0.825859	0.845711	0.864977	0.818913	0.834036
Raw Coverage	0.820484	0.639166	0.350379	0.647312	0.751977	0.761662	0.590103	0.791058	0.796888
Unique Coverage	0.225833	0.044514	0.103332	0.400265	0.100925	0.11061	0.590103	0.086436	0.092266
Overall Consistency	0.820782		0.850549		0.810128		0.864977	0.781986	
Overall Coverage	0.864999		0.750644		0.862587		0.590103	0.883325	
Consistency Threshold	0.869762		0.857448		0.857851		0.864977	0.862817	
Outcome: Compatibility			Outcome: Extent		Outcome: Mentally away		Outcome: Physically away	Outcome: Fascination	

Note: “⊗” indicates the absence of a core condition; “●” indicates the presence of a core condition; A blank cell signifies that the condition may be either present or absent without affecting the outcome.

For Compatibility, two equifinal configurations emerge, with an overall consistency of 0.821 and coverage of 0.865, indicating high explanatory power. Extent also exhibits two sufficient pathways (overall consistency = 0.851; coverage = 0.751). Likewise, Mentally away is produced by two configurations (consistency = 0.810; coverage = 0.863). Willingness to physically away yields a single sufficient configuration (consistency = 0.865; coverage = 0.590). Finally, fascination is covered by one configuration (consistency = 0.782; coverage = 0.883). In sum, all models demonstrate satisfactory fit and substantive explanatory capacity.

Configuration paths with Compatibility as the output variable: Configuration 1 has high environmental preference as its core condition. This configuration path indicates that when an individual’s environmental preference is high, their compatibility value also increases. Configuration 2 has high natural environment perception and high environmental preference as its core conditions. This configuration path indicates that when an individual’s natural environment perception and environmental preference are both high, their compatibility value also increases.

Configuration paths with extent as the output variable: Configuration 1 has natural environment perception as its core condition. This type of configuration path indicates that when an individual’s natural environment perception is high, their extent will also be high. Configuration 2 has high natural environment perception and high place attachment as its core conditions. This type of configuration path indicates that when an individual’s natural environment perception and place attachment are both high, their Extent will also be high.

For configuration paths with Mentally away as the output variable: Configuration 1 has natural environment perception as its core condition. This type of configuration path indicates that when an individual’s the natural environment perception is high, their mental away is also high. Configuration 2 has high place attachment as its core condition. This type of configuration path indicates that when an individual’s place attachment is high, their Mentally away is also high.

For configuration paths with physically away as the output variable, the core conditions are natural environment perception, environmental preference, and place attachment. This configuration path indicates that when an individual has high natural environment perception, environmental preference, and place attachment, their Physically away will also be high.

For configuration paths with fascination as the output variable: Configuration 1 has natural environment perception as its core condition. This configuration path indicates that when an individual’s natural environment perception is high, their fascination will also be high. Configuration 2 has high environmental preference as its core condition. This configuration path indicates that when an individual’s environmental preference is high, their Fascination will also be high.

## 5. Discussion

The ecological benefits and economic value of forest parks are extremely high. Forest parks are also rich in psychological and physiological resources, in addition to their recreational and educational functions. It is crucial to pay attention to the impact of the natural environment of forest parks on human psychology. Structural equation modeling is utilized in this study to explore the relationship between the natural environment and restorative environmental perception, and to verify the mechanism and path of the natural environment’s influence on restorative environmental perception. The results showed that 22 out of the 33 pre-proposed path relationships were valid. The results are being discussed and analyzed in the following.

The findings unequivocally identify Environmental Preference as a cognitive ‘gatekeeper’ in the restorative process. Unlike the Stress Reduction Theory which emphasizes automatic physiological responses, our results suggest that for the Zhangjiajie tourist, restoration is cognitively mediated. The sensory input of the natural environment perception must first pass through an evaluative filter—’Do I like this?’—before it can trigger the deeper dimensions of fascination and being away. If this aesthetic evaluation is negative, the restorative chain is effectively blocked, regardless of the objective quality of the nature.

The natural environment perception exerts a significant and positive influence on individuals’ restorative perception. Humans possess an innate connection with nature that not only supplies material necessities for survival but also harbours vital psychological resources indispensable for modern life and essential to maintaining physical and mental health [98]. Accumulating evidence indicates that natural environments are valued not merely for their intrinsic worth but also for their recreational functions [99] and, more importantly, as a critical resource for promoting psychological and physiological well-being [100–103]. Even brief encounters with nature or passive observation of surrounding natural scenery can effectively alleviate mental fatigue. The present findings corroborate the restorative capacity of natural environments: natural environment perception significantly enhances both the “Mentally away” and “fascination” dimensions of restorative experience, whereas its effects on compatibility, extent, and Physically away remain non-significant.

Natural environments inherently provide the quality of “being away,” enabling individuals to detach from daily stressors and fatigue and thereby replenish their psychological resources. Attention Restoration Theory (ART) posits that this sense of separation is essential for psychological recovery [104]. The captivating and novel features of natural settings capture individuals’ involuntary attention, fostering deep immersion and a sense of fascination that not only facilitates restoration but also exerts a significant positive effect on well-being [104]. Compatibility refers to the congruence between environmental affordances and individuals’ needs. Although natural environments are generally perceived as highly compatible, situational factors—such as temperature or humidity falling outside an individual’s comfort range—may attenuate this effect [105]. Extent denotes the perceived scope or richness of the restorative environment; while natural settings typically offer high restorative potential, the perceived intensity may sometimes be insufficient to elicit a significant restorative response when individuals’engagement with the environment is low. “Physically away” describes objective distance from everyday surroundings. Although physical separation can contribute to restoration, its impact is contingent upon concurrent psychological detachment; if the mind remains preoccupied, the restorative benefits of physical relocation are markedly diminished [106].

The results reveal that environmental preference fully mediates the relationship between natural-environment perception and restorative perceptions. Although natural environment perception exerts direct and significant effects only on the Mentally away and Fascination dimensions of restorative perception, the mediating role of environmental preference amplifies and extends its influence to four of the five restorative dimensions—namely Compatibility, Extent, Fascination, and Mentally away—while excluding Physically away. Environmental preference thus functions as an automatic appraisal mechanism through which individuals rapidly determine whether a given setting is worthy of approach or avoidance, thereby translating initial perceptions of nature into broader restorative experiences [11,107,108].Individuals’ preference for natural environments is shaped by their perception of these settings’ superior restorative potential. For environmental preference to function as a mediator (or moderator), three conditions must be satisfied simultaneously: (1) a significant association between the type of environment and preference; (2) a significant association between the type of environment and perceived restorative potential; and (3) a statistically significant association between restorative potential and preference when the influence of environment type on preference is held constant. Only when all three conditions are met can environmental preference effectively exert its mediating influence [109,110]. Contrary to these theoretical claims, our results indicate that place attachment does not significantly mediate the relationship between natural-environment perception and restorative perception. This finding diverges from prior assertions that the restorative quality of highly cherished settings stems from their capacity to offer a secure and comfortable context, thereby enabling self-regulation, stress reduction, and the redirection of attention toward problem-solving and self-reflection—processes that ultimately help individuals organize their thoughts and emotions [111]. This non-significant effect may be attributable to the multidimensional nature of place attachment. Its two core dimensions—place dependence and place identity—can exert divergent influences on perceived restoration, while natural-environment perception and restorative perception themselves retain a degree of conceptual and empirical independence. Certain environmental qualities may therefore impinge directly on restorative outcomes without necessitating the involvement of place attachment as an intermediary. Additionally, contextual characteristics, individual differences, and limitations inherent to research design and measurement instruments may further constrain the mediating capacity of place attachment. Future studies should disentangle the distinct roles of place-dependence and place-identity across varied environmental settings and individual profiles, thereby yielding a more nuanced understanding of this phenomenon [27,112]. Future research should therefore delve into the distinct mechanisms underlying the dimensions of place attachment and examine their mediating effects across diverse environmental contexts and individual profiles, thereby providing a more comprehensive understanding of this phenomenon. The present study further reveals that environmental preference and place attachment jointly operate as a significant serial mediator between natural-environment perception and restorative perception. This sequential effect likely arises because environmental preference functions as an initial bridge, intensifying individuals’ emotional bonds with a specific setting and thereby fostering the formation of place attachment [113]. Once established, place attachment deepens psychological investment in the environment, motivating individuals to actively seek restorative experiences within that locale. Such heightened engagement facilitates the recognition of restorative qualities, thereby elevating overall restorative perceptions [114]. Moreover, a serial mediation pathway can accumulate the effects of multiple mediators, amplifying the influence of natural environment perception on restorative perception [115]. Consequently, the significant serial mediation of environmental preference and place attachment is attributable to the former’s role in strengthening emotional ties and the latter’s role in deepening psychological commitment, jointly enhancing restorative experiences and, ultimately, restorative perceptions.

This study finds that place attachment positively impacts certain dimensions of the restorative environmental perception, specifically compatibility and degree, aligning with some prior research. Yang (2018) [77] highlighted a strong correlation between place attachment to one’s origin and restorative perception, noting that stronger attachment enhances restorative experiences in familiar environments. Chi & Su (2012) [76] showed that attachment to an environment affects the restorative impact of natural scenes on emotional and attentional states, with only attached environments providing restorative benefits. Conversely, low attachment can lead to negative emotional activation, hindering psychological recovery. Feng & Cui (2015) [116] found that high-quality urban environments foster restorative experiences, thereby strengthening place attachment, with physical environment quality being a key predictor. Place attachment aids self-regulation by reducing cognitive load and enhancing positive emotions, supporting goal attainment [117–119]. Favorite places, which are often sites of strong attachment, facilitate self-reflection and stress relief, crucial for self-regulation [25]. Wang & Zheng (2015) [120] observed that emotional restoration in green spaces was more significant for individuals from grassland origins, suggesting that origin environments influence restorative perceptions. Liu et al. (2017) [121] and Peng (2017) [122] explored the interplay between place attachment and restorative perception in recreational and community park settings, revealing that place attachment and environmental characteristics jointly influence restorative effects. However, this study’s findings differ from Xi et al. (2021) [27], who reported broader impacts of place attachment on restorative perception in resort settings, possibly due to differences in study sites. Future research could compare natural and man-made environments to further elucidate these dynamics.

According to Liu et al. (2019) [68], environmental preference has a significant and positive impact on four dimensions of restorative perception except physically away, and environmental preference has a significant and positive impact on place attachment. Individuals’ preferred place environments can promote the emergence of a sense of attachment to the environment and the development of individual emotions, thus triggering an individual’s emotional connection to the environment, generating a sense of place identity, and further contributing to the individual’s acquisition of a restorative experience. The environmental preference has a significant and positive impact on place attachment; the more favorable the place environment is, the more attachment is created, which is consistent with Zhu (2020)’s findings [123]. The study also confirmed that environmental preferences play a partial mediating role between perceptions of the natural environment and perceptions of the restorative environment. Perceptions of the natural environment influenced the degree dimension of the restorative environmental perception through the chain mediation of environmental preferences and place attachment. This is in accordance with the findings of Faraji et al. (2024) [124].

The PLS-SEM findings largely converge with those of the fsQCA. Environmental preference exerts a significant mediating effect and consistently appears as a core condition across alternative configurational paths in the fsQCA, whereas the mediating role of place attachment is nonsignificant. Natural environment perception demonstrates a pronounced positive effect on both mental away and fascination in the PLS-SEM and is likewise identified as a core condition in the fsQCA solutions. Complementing the PLS-SEM results, the fsQCA not only corroborates the symmetric relationships uncovered but also offers a more holistic and nuanced understanding of the antecedents of the restorative environmental perception through configurational analysis.

Contrary to Hypothesis H24-H28, PLS-SEM results showed that place attachment alone did not significantly mediate the relationship between natural environment perception and restorative environmental perception. This finding diverges from studies emphasizing the restorative role of “favorite places”. However, the fsQCA results provide a nuanced explanation: place attachment appears as a peripheral condition in high-restoration configurations.

This suggests that for the typical tourist in Zhangjiajie (often a first-time visitor), restoration is primarily driven by the immediate sensory impact of the natural environment perception. The formation of place attachment typically requires repeated interaction and time, which may not be present for all tourists. Thus, while place attachment can enhance restoration (as shown in the serial mediation path), it is not a necessary condition. High restoration can occur purely through strong nature perception and preference, even in the absence of deep attachment. This distinction between “core” (environmental preference) and “peripheral” (place attachment) factors is a key theoretical contribution of this study, refining our understanding of how restoration occurs in tourism contexts compared to residential contexts.

A notable limitation of this study is the seasonality of data collection. The survey was conducted in June, a period characterized by lush vegetation and vibrant natural scenery in Zhangjiajie. According to Pratiwi et al. (2019) [125], landscape imagery and restorative potential can vary significantly across seasons. Winter landscapes, for instance, might evoke different levels of fascination or compatibility due to reduced greenery. Therefore, our findings primarily reflect the ‘summer mechanism’ of restoration. Future studies should adopt a longitudinal approach to examine how seasonal changes in natural environment perception moderate the restorative process.

## 6. Conclusion

In this study, we constructed a model of the relationship between natural environment perception, environmental preference, place attachment, and restorative environmental perception, and proposed 33 research hypotheses. At the same time, Zhangjiajie National Forest Park was selected as a case study, and the data were obtained through a questionnaire survey and analyzed by Smart PLS 4.0 and fsQCA 3.0. The following conclusions were drawn: (1) Two dimensions of restorative environmental perception (mentally away and fascination) are significantly influenced by natural environment perception. The more tourists perceive the natural environment subjectively, the greater their mentally away and fascination. (2) Environmental preference is significantly influenced by natural environment perception. The more tourists perceive the natural environment subjectively, the more they tend to prefer it. (3) The relationship be-tween natural environment perception and restorative environmental perception was significantly mediated by environmental preference. However, the mediating effect of place attachment alone was not significant. In contrast, the chain mediation effect of environmental preference and place attachment was significant. Additionally, fsQCA was employed to complement the symmetric findings of PLS-SEM. The configurational analysis confirmed that environmental preference consistently functioned as a core condition across multiple pathways leading to high levels of restorative perception outcomes. Natural environment perception also emerged as a core condition in nearly all high-outcome configurations, while place attachment played a more conditional and peripheral role. These findings reinforce the mediating dominance of environmental preference and provide a more nuanced, asymmetric understanding of how combinations of conditions influence tourists’ restorative experiences.

## Supporting information

S1 File Data set. (XLSX)
